# Association Between Edmonton Obesity Staging System Severity and 90-Day Postoperative Complications After Primary Metabolic and Bariatric Surgery: A Retrospective Cohort Study

**DOI:** 10.1007/s11695-026-08759-2

**Published:** 2026-06-03

**Authors:** Anna Carolina Batista Dantas, Denis Pajecki, Priscila Caproni, Beatriz Helena Tess, Marco Aurelio Santo

**Affiliations:** 1 Unidade de Cirurgia Bariátrica E Metabólica, Disciplina de Cirurgia Do Aparelho Digestivo E Coloproctologia, Departamento de Gastroenterologia, Hospital das Clínicas HCFMUSP, Faculdade de Medicina, Universidade de São Paulo, São Paulo, Brazil; 2 Departamento de Cirurgia, Universidade Federal do Rio Grande do Norte, Natal, Brazil; 3 Departamento de Medicina Preventiva, Faculdade de Medicina, Universidade de São Paulo, São Paulo, Brazil

**Keywords:** Obesity, Bariatric Surgery, Postoperative Complications, Disease Staging, Risk Assessment, Perioperative Care

## Abstract

**Introduction:**

Obesity is a chronic multifactorial disease, and the Edmonton Obesity Staging System (EOSS) has been proposed as a tool to grade disease severity and estimate perioperative risk in patients undergoing metabolic and bariatric surgery (MBS). However, its association with postoperative complications in those with more advanced stages of obesity remains insufficiently explored.

**Objective:**

To assess the association between EOSS severity with 90-day postoperative complications after MBS.

**Methods:**

This retrospective cohort study included patients who underwent primary laparoscopic sleeve gastrectomy (SG) or Roux-en-Y gastric bypass (RYGB) between January 2020 and December 2023. Epidemiological and clinical data, EOSS classification, and 90-day postoperative complications were collected. Complications were classified according to the Clavien–Dindo grading system. Univariate and multivariable logistic regression analyses were performed.

**Results:**

A total of 335 patients were included, with a median age of 47 years (IQR 39–56), 81.8% were female, and the median body mass index (BMI) was 44.9 kg/m² (IQR 40.8–49.4). The prevalence of EOSS stages 3–4 was 33.4%. A significant difference was observed in the distribution of surgical techniques (*p* = 0.002), with an increase in SG use across more advanced EOSS. Major 90-day complication rate was 5.4% (*n* = 18), ranging from 1.8% in patients with EOSS 0–2 to 12.5% in those with EOSS 3–4 (*p* < 0.001). The reoperation rate was 1.5%, and no deaths occurred. Exploratory multivariable logistic regression models showed that higher EOSS stages were independently associated with increased odds of both overall complications (OR 1.90; 95% CI 1.30–2.80; *p* = 0.001) and major complications (OR 4.16; 95% CI 1.83–11.0; *p* = 0.0015).

**Conclusion:**

Advanced EOSS stages were associated with higher 90-day postoperative complication rates after MBS in this cohort of patients with severe obesity and substantial disease burden. While EOSS may help identify patients at increased perioperative risk, it should not be interpreted as an isolated predictor of surgical morbidity.

**Supplementary Information:**

The online version contains supplementary material available at 10.1007/s11695-026-08759-2.

## Keypoints

Advanced EOSS stages are associated with a significantly higher rate of overall and major complications within 90 days after metabolic and bariatric surgery.

Patients with EOSS 3–4 represent a higher-risk subgroup, with a marked increase in complication rates compared to those with early-stage disease.

EOSS is a clinically useful tool for risk stratification but should not be used as a sole predictor of surgical morbidity.

**Supplementary Information:**

The online version contains supplementary material available at 10.1007/s11695-026-08759-2.

## Introduction

Metabolic and bariatric surgery (MBS) has emerged as a safe, effective, and durable treatment for severe obesity and its related complications [1, 2]. In recent years, growing evidence has demonstrated that MBS can be safely performed in patients with advanced disease and end-organ damage, including those with end-stage kidney disease secondary to type 2 diabetes (T2D), heart failure, large abdominal wall hernias, and elderly patients [3–6].

Historically, early postoperative morbidity has been associated with higher body mass index (BMI), advanced age, and male gender [7, 8]. However, as MBS is increasingly performed in patients with greater complexity, there is a growing need for more comprehensive risk-stratification tools [9]. The Edmonton Obesity Staging System (EOSS) was developed to classify obesity severity based on clinical, functional, and mental health domains. Initially proposed to guide treatment prioritization, EOSS has subsequently been validated as a predictor of mortality and postoperative morbidity [10–12].

Although several studies have examined the association between EOSS and perioperative outcomes after MBS, demonstrating increased complication rates in more advanced stages, these analyses have been limited by the low prevalence of EOSS 3–4 [12, 13]. In the seminal study by Chiappetta et al., complication rates increased with EOSS severity; however, only a small proportion of patients were classified as stage 3, and EOSS 4 was nearly absent [14]. Similarly, subsequent studies, including those from the original Edmonton group, confirmed an association between EOSS stage 3 and a higher risk of major complications, yet included a limited number of patients with advanced disease [15]. This restricted distribution limits the ability to fully evaluate the prognostic performance of EOSS in the most complex population, in whom disease burden extends beyond isolated obesity-related medical problems to include end-organ damage, functional impairment, and psychological morbidity. Therefore, the role of EOSS as a risk stratification tool in patients with advanced obesity remains uncertain.

The present study aimed to evaluate the association between EOSS severity and 90-day postoperative complications following MBS in a cohort with a high prevalence of advanced-stage disease.

## Methods

### Study Design

This retrospective cohort study was conducted at a quaternary academic public hospital in Brazil. The study included adult patients who underwent primary MBS between January 2020 and December 2023. Postoperative outcomes were assessed within 90 days after surgery. Data were obtained from a prospectively maintained institutional MBS registry and verified through a comprehensive retrospective review of electronic health records (EHR). Data extraction and verification were performed during 2024. All patient information was anonymized, and the study was approved by the local Research Ethics Committee. The study was designed and reported in accordance with the Strengthening the Reporting of Observational Studies in Epidemiology (STROBE) guidelines [16].

### Study Population

The study population consisted of consecutive adult patients (≥ 18 years) who underwent primary MBS with either sleeve gastrectomy (SG) or Roux-en-Y gastric bypass (RYGB) during the study period. Patients were excluded if they had undergone revisional, conversional, or staged bariatric procedures, or if perioperative data required for analysis were missing or incomplete. Postoperative outcomes were assessed through review of EHR and routine outpatient clinic follow-up visits, allowing identification of complications occurring within the 90-day postoperative period.

### Variables and Data Sources

All clinical, operative, and postoperative variables were obtained through a combination of automated extraction from the institutional MBS registry and manual review of the EHR. Data were collected and managed using REDCap (Research Electronic Data Capture) [17]. Data abstraction, including EOSS staging, was performed independently by two investigators using a standardized data collection form and predefined criteria (Supplement 1A), with any discrepancies or uncertainties reviewed jointly and resolved by consensus. Given the absence of persistent disagreements, formal inter-rater reliability metrics were not calculated.

Baseline variables included demographic characteristics (age and gender), anthropometric data (BMI), obesity-related medical problems, and relevant medical history. The diagnoses and severity of the conditions were established based on EHR documentation and, when available, specialist assessments. The primary outcomes were overall postoperative complications and major complications occurring within 90 days after surgery. Major complications were defined as Clavien–Dindo grade ≥ IIIa [18]. Secondary outcomes included hospital readmission, reoperation, and mortality within the same postoperative period. Emergency department visits were recorded as part of postoperative follow-up. Events were classified as complications only when a clinical diagnosis requiring evaluation or treatment was established and subsequently graded according to the Clavien–Dindo classification. All hospital readmissions in this cohort were related to postoperative complications and are included in the reported complication rates.

Obesity severity was assessed using the EOSS, and each patient was retrospectively classified according to published criteria [10]. In this framework, stage 0 corresponds to the absence of obesity-related clinical, mental, or functional impairments. Stage 1 reflects the presence of subclinical risk factors or mild symptoms without established chronic disease. Stage 2 includes patients with established obesity-related chronic conditions requiring medical treatment, such as hypertension, type 2 diabetes, obstructive sleep apnea, osteoarthritis, or gastroesophageal reflux disease. Stage 3 is characterized by end-organ damage or significant clinical complications of obesity-related disease, including cardiovascular or renal complications, as well as marked functional limitations or significant psychopathology. Stage 4 represents severe disability or end-stage complications associated with obesity-related disease. EOSS staging was assigned retrospectively through structured electronic health record review using predefined criteria based on the original EOSS framework (Supplement 1A). Obesity-related clinical, functional, and mental health variables were individually collected using a standardized REDCap form and subsequently categorized according to their respective EOSS domain severity scores. Clinical, mental, and functional domains were evaluated separately, and the highest score within each domain was retained. The final EOSS stage corresponded to the highest severity score identified across all domains. For analytical purposes, patients were grouped into early (EOSS 0–2) and advanced (EOSS 3–4) stages. This categorization was based on prior literature and on the conceptual distinction between stages without end-organ damage (EOSS 0–2) and those characterized by established organ dysfunction or significant functional impairment (EOSS 3–4), which have been consistently associated with higher perioperative risk and mortality [11, 14].

### Statistical Analysis

Continuous variables were assessed for normality using the Shapiro–Wilk test. Normally distributed variables were reported as mean ± standard deviation, whereas non-normally distributed variables were expressed as median and interquartile range (IQR). Categorical variables were presented as absolute frequencies and percentages. Comparisons between groups were performed using the Student’s t-test or Mann–Whitney U test for continuous variables, as appropriate, and the chi-square test or Fisher’s exact test for categorical variables.

EOSS was analyzed both as an ordinal variable and as grouped stages (0–2 vs. 3–4). Binary logistic regression models were used to evaluate factors associated with (1) overall postoperative complications and (2) major complications (Clavien–Dindo ≥ IIIa) within 90 days. Initially, univariate analyses were performed to explore associations between individual variables and outcomes. Variables with p-value < 0.20 in univariate analysis were selected for inclusion in the multivariable models.

To address potential collinearity between EOSS and its individual components, two separate multivariable modeling strategies were applied. Model A included EOSS along with demographic and surgical variables (age, sex, BMI, and procedure type) to evaluate its association with outcomes. Model B included individual obesity-related medical problems without EOSS to explore the contribution of specific clinical factors. Given the conceptual overlap between EOSS and its component variables, the EOSS-based model (Model A) was considered the primary analytical approach, as it reflects the integrated disease severity construct evaluated in this study.

Because major complications were infrequent, with only 18 events, the multivariable analysis for major complications was deliberately kept parsimonious to reduce the risk of overfitting. A multivariable logistic regression model was initially constructed using clinically relevant candidate predictors. Backward selection was then used to obtain a parsimonious final model, retaining EOSS stage as the main predictor of interest and procedure type as a clinically relevant adjustment variable. The final model for major complications therefore included EOSS stage and procedure type. To reduce the risk of overfitting, particular attention was given to the number of events per variable included in each model. Multicollinearity was assessed using variance inflation factors (VIFs). Adjusted odds ratios (aORs) with 95% confidence intervals (CIs) were reported. Model discrimination for the final major-complication model was assessed using the receiver operating characteristic curve and area under the curve (AUC). Overall model fit was evaluated using likelihood ratio testing. These analyses are presented in the Supplementary Material.

No cases were excluded due to missing data, as complete information was available for all variables included in the analyses. No formal sensitivity analyses were performed. A two-sided p-value < 0.05 was considered statistically significant. All statistical analyses were performed in R (version 4.5.1; R Foundation for Statistical Computing, Vienna, Austria) using RStudio.

Artificial intelligence (AI) tools were used to a limited extent for language editing, formatting, and data analysis support. All analytical decisions, results interpretation, and scientific content were determined by the authors.

## Results

### Study Population

Between January 2020 and December 2023, 383 patients underwent MBS at our institution. After excluding revisional procedures (*n* = 25), staged procedures (*n* = 1), and surgeries using techniques not included in the study (One Anastomosis Gastric Bypass *n* = 13; SG with transit bipartition, *n* = 9), 335 patients were included in the final analysis (Supplement 1B).

The cohort had a median age of 47 years (IQR 39–56), 81.8% were female, and the median BMI was 44.9 kg/m² (IQR 40.8–49.4). The most prevalent associated medical problems were Metabolic Dysfunction-Associated Steatotic Liver Disease (MASLD) (75.8%), hypertension (65.7%), T2D (44.2%), dyslipidemia (36.7%), and obstructive sleep apnea (OSA) (31.6%), as shown in Table 1. RYGB was the most frequently performed procedure (67.8%), followed by SG (31.9%). The distribution of EOSS stages was as follows: 0.9% (stage 0), 3.9% (stage 1), 61.8% (stage 2), 27.8% (stage 3), and 5.7% (stage 4), with 33.4% of patients classified as EOSS 3–4.

**Table 1:** Demographic and clinical characteristics of all patients according to EOSS stages

		Total	EOSS 0	EOSS 1	EOSS 2	EOSS 3	EOSS 4	*p* value
Sample, *N* (%)		335	3 (0.9)	13 (3.9)	207 (61.8)	93 (27.8)	19 (5.7)	
Age*, years		47.0 (39.0–56.0)	29 (28–33.5)	39 (36–44)	47 (40–55.5)	48 (41–59)	50 (38–58.5)	**0.001** ^a^
Gender, *n* (%)								**0.004** ^b^
	Female	274 (81.8)	3 (100)	13 (100)	179 (86.5)	67 (72)	12 (63.2)	
	Male	61 (18.2)	0	0	28 (13.5)	26 (28)	7 (36.8)	
Weight*, kg		118 (108–135)	136 (135.5–140.5)	133 (125–150)	130 (120–150)	130 (112–149)	132 (124–157.5)	0.557 ^a^
BMI*, kg/m^2^		44.9 (40.8–49.4)	41.5 (41.3–42.5)	48.4 (46.2–52.5)	45.5 (41.1–49.7)	41.9 (38.5–48.3)	44.7 (43–49.2)	**0.003** ^a^
Obesity-related clinical diseases								
T2D, *n* (%)		148 (44.2)	-	-	92 (44.4)	49 (52.7)	7 (36.8)	**0.002** ^b^
Hypertension, *n* (%)		220 (65.7)	-	-	136 (65.7)	68 (73.1)	16 (84.2)	**< 0.001** ^b^
OSA, *n* (%)		106 (31.6)	-	-	57 (27.5)	41 (41.1)	8 (42.1)	**0.033** ^b^
Dyslipidemia, *n* (%)		123 (36.7)	-	-	63 (30.4)	49 (52.7)	11 (57.9)	**0.004** ^b^
MASLD, *n* (%)		254 (75.8)	-	-	177 (85.5)	67 (72)	10 (52.6)	**< 0.001** ^b^
Kidney disease, *n* (%)		13 (3.9)	-	-	1 (0.5)	8 (8.6)	4 (21.1)	**0.009** ^b^
Alcohol use, *n* (%)		25 (7.5)	0	0	14 (6.8)	9 (9.7)	2 (10.5)	0.663 ^b^
Smoking, *n* (%)		53 (15.8)	0	2 (15.4)	28 (13.5)	18 (19.4)	5 (26.3)	0.349 ^b^
Cancer history, *n* (%)		19 (5.7)	0	0	13 (6.3)	5 (5.4)	1 (5.3)	0.893 ^b^
Use of anticoagulation, *n* (%)		12 (3.6)	0	0	1 (0.5)	6 (6.5)	5 (26.3)	**< 0.001** ^b^
Use of corticosteroids, *n* (%)		4 (1.2)	0	0	0	1 (1.1)	3 (15.8)	**0.001** ^b^
Bariatric procedure, *n* (%)								**0.002** ^b^
	RYGB	228 (67.8)	3 (100)	12 (92.3)	149 (72)	57 (61.3)	7 (36.8)	
	SG	107 (31.9)	0	1 (7.7)	58 (28)	36 (38.7)	12 (63.2)	

^a^Mann–Whitney U test; ^b^Pearson’s chi-square test

*Data are presented as median (Q1–Q3)

*BMI *body mass index, *T2D *type 2 diabetes, *OSA *obstructive sleep apnea, *MASLD *metabolic dysfunction-associated steatotic liver disease, *RYGB *roux-en-y gastric bypass, *SG *sleeve gastrectomy

**Fig. 1:**
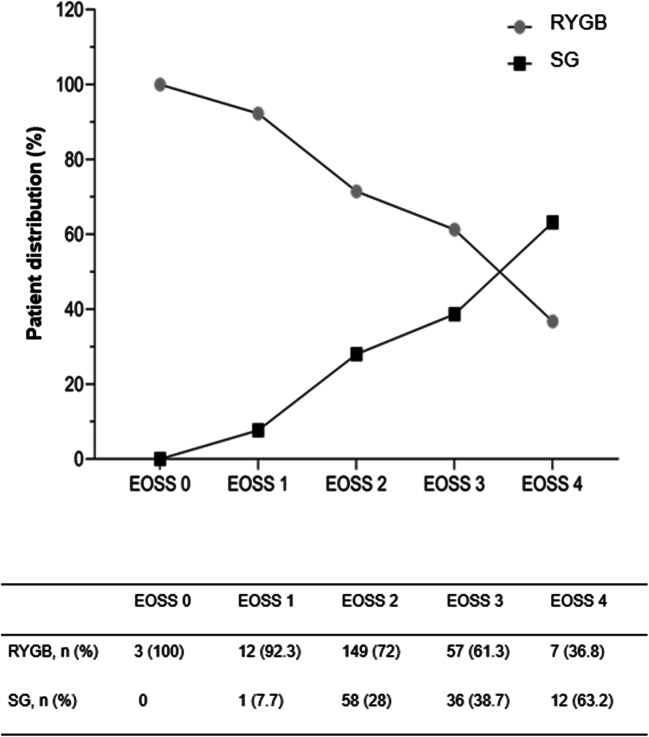
Distribution of bariatric procedures according to EOSS

Obesity-related medical problems patterns differed significantly across EOSS stages. T2D (*p* = 0.002) and MASLD (*p* < 0.001) were more prevalent in EOSS 2 and 3 and less frequent in EOSS 4, whereas hypertension (*p* < 0.001), OSA (*p* = 0.033), and dyslipidemia (*p* = 0.004) showed aa increase in prevalence with advancing EOSS stages. A significant difference in surgical technique distribution was observed across EOSS stages (*p* = 0.002), with an increase in the proportion of SG in more advanced stages, reaching 63.2% in EOSS 4 (Fig. 1).

### Postoperative outcomes

The overall rate of postoperative complications within 90 days was 27.8% (*n* = 93), while major complications occurred in 5.4% of patients (*n* = 18). The reoperation rate was 1.5%, and there was no mortality.

Complications occurred at a median of 10 days after surgery (IQR 3–39). Readmission occurred in 5.7% of patients, and 12.5% sought emergency care during the postoperative period. According to the Clavien–Dindo classification, most complications were minor, with grade I events occurring in 5.9% of patients and grade II in 16.4%. Major complications were less frequent, including 0.9% grade IIIa, no grade IIIb events, 2.4% grade IVa, and 2.1% grade IVb. No grade V events were observed, as detailed in Table 2.

**Table 2:** Characteristics of 90-day postoperative complications

		*N* = 93
Postoperative time, median (Q1 - Q3), days		10 (3–39)
Emergency care, *n* (%)		42 (12.5)
Hospital readmission, *n* (%)		19 (5.7)
Reoperation, *n* (%)		5 (1.5)
Mortality, *n* (%)		0
**Clavien-Dindo classification**,***n*** **(%)**		
	I	20 (5.9)
	II	55 (16.4)
	IIIa	3 (0.9)
	IIIb	0
	IVa	8 (2.4)
	IVb	7 (2.1)
	V	0

Among grade I complications, the most frequent were food intolerance, gastrointestinal bleeding, abdominal bleeding, and abdominal pain (*n* = 3 each), all managed conservatively. Grade II complications were more common and predominantly included acute kidney injury (*n* = 9), constipation (*n* = 7), food intolerance (*n* = 7), abdominal pain (*n* = 7), and superficial surgical site infections (*n* = 6) managed with medical treatment. Grade III complications were infrequent and consisted of gastrojejunal stenosis and bleeding requiring endoscopic therapy (*n* = 3). Grade IV complications included severe events requiring ICU management, most commonly hemorrhagic shock due to abdominal or gastrointestinal bleeding (*n* = 5) and septic shock (*n* = 2), as detailed in Table 3.

**Table 3:** 90-day postoperative complications according to Clavien-Dindo grading system

	*N* = 335
**Grade I**	***N*** **=20 (5.9%)**
Food intolerance (investigated with UGI and/or UGE, managed with dietary changes)	3
GI bleeding (melena diagnosed clinically, managed conservatively)	3
Abdominal bleeding (investigated with abdominal CT, managed conservatively)	3
Abdominal pain (investigated with abdominal CT, managed conservatively)	3
Respiratory symptoms (not COVID, managed conservatively)	2
Fall due to orthostatic hypotension (head CT, managed conservatively)	2
Intraoperative positive methylene blue testing (early postoperative UGI)	2
Superficial wound dehiscence (managed conservatively with dressing changes)	1
Hypotension during hemodialysis (managed conservatively)	1
**Grade II**	***N*** **= 55 (16.4%)**
AKI (managed with IV fluids and supportive care)	9
Constipation (managed with laxative medication)	7
Food intolerance (investigated with UGI and/or UGE, managed with medication and oral nutritional support)	7
Abdominal pain (investigated with abdominal CT, managed with pain medication)	7
Superficial SSI (managed with bedside incision and drainage, oral antibiotics)	6
Nausea and vomiting (managed with IV medication and fluid resuscitation)	4
AKI on CKD (managed with IV fluids and supportive care)	3
UTI (managed with oral antibiotics)	3
Bronchospasm (managed with pharmacologic therapy and supplemental oxygen)	3
Abdominal bleeding (investigated with abdominal CT, managed with blood transfusion)	2
Marginal ulcer (diagnosed with UGE and managed conservatively with PPIs)	2
Gastrojejunal anastomotic leak (diagnosed with abdominal CT and managed with IV antibiotics)	1
Symptomatic B12 deficiency (managed with intramuscular cyanocobalamin)	1
**Grade III**	***N*** **= 3 (0.9%)**
Gastrojejunal stenosis (managed with endoscopic dilation)	2
Gastrojejunal bleeding (managed with UGE therapy)	1
**Grade IV**	***N*** **= 15 (4**,**5%)**
Abdominal bleeding (hemorrhagic shock managed in ICU with 2 reoperation)	3
GI bleeding (hemorrhagic shock managed in ICU with blood transfusion)	2
Septic shock (IV antibiotics and surgery for pressure ulcer and small bowel injury)	2
AKI on CKD (managed in ICU, but without dialysis)	2
Pulmonary embolism (managed in ICU with anticoagulation and supplemental oxygen)	1
Tension pneumothorax(managed in ICU due to intraoperative hemodynamic instability)	1
Pneumonia (managed in ICU with IV antibiotics and supplemental oxygen)	1
Hypertensive crisis (managed with vasoactive therapy in the ICU)	1
Acute pulmonary edema due to COVID-19 (Medical management in the ICU)	1
Decompensated heart failure (managed in ICU with vasoactive support)	1

Data is presented as frequency and percentage (%)

*UGI* upper gastrointestinal image, *UGE *upper gastrointestinal endoscopy, *GI *gastrointestinal, *CT *computed tomography, *COVID *coronavirus disease, *AKI *acute kidney injury, *IV *intravenous, *SSI *surgical site infection, *CKD *chronic kidney disease, *UTI *urinary tract infection, *PPI *proton pump inhibitor, *ICU *intensive care unit

Among patients who developed postoperative complications, 75 (80.6%) were classified as minor and 18 (19.4%) as major. Patients with major complications had a higher prevalence of OSA compared to those with minor complications (66.7% vs. 26.6%; *p* < 0.001) and were more frequently classified as EOSS 3–4 (77.8% vs. 42.7%; *p* = 0.009). Time to event also differed significantly, with major complications occurring earlier (median 2.5 days; IQR 1.0–6.0) compared to minor complications (median 12.0 days; IQR 5.0–41.0; *p* = 0.001). No significant differences were observed between groups for other demographic, clinical, or surgical variables (Table 4).

**Table 4:** Demographic and clinical characteristics according to complication severity

		Minor complication	Major complication	*p* value
*N *(%)		75 (80.6)	18 (19.4)	
Age, median (Q1-Q3), years		46.0 (40.0–59.5)	53.5 (47.2–63.2)	0.118 ^a^
Gender, *n* (%)				0.093 ^b^
	Female	62 (82.7)	11 (61.1)	
	Male	13 (17.3)	7 (38.9)	
Weight, median (Q1-Q3), kg		116.4 (105.5–130.0)	118.5 (98.3–136.0)	0.973 ^a^
BMI, median (Q1-Q3), kg/m^2^		43.7 (40.7–49.9)	43.5 (38.5–46.1)	0.408 ^a^
T2D, *n* (%)		35 (46.7)	12 (66.7)	0.279 ^b^
Insulin use, *n* (%)		13 (37.1)	8 (66.7)	0.100 ^b^
Hypertension, *n* (%)		52 (69.3)	16 (88.9)	0.139 ^b^
OSA, *n *(%)		20 (26.6)	12 (66.7)	**< 0.001** ^b^
Dyslipidemia, *n* (%)		29 (38.7)	10 (55.5)	0.404 ^b^
MASLD, *n* (%)		55 (75.3)	11 (61.1)	0.359 ^b^
Kidney disease, *n* (%)		4 (5.3)	2 (11.1)	0.328 ^b^
Alcohol use, *n* (%)		5 (6.7)	3 (16.6)	0.181 ^b^
Smoking, *n* (%)		9 (12.0)	5 (29.4)	0.153 ^b^
Cancer history, *n* (%)		4 (5.3)	1 (5.6)	1.000 ^b^
Use of anticoagulation, *n* (%)		2 (3.1)	3 (16.7)	0.068 ^b^
Use of corticosteroids, *n* (%)		1 (1.6)	2 (11.1)	0.328 ^b^
Bariatric procedure, *n* (%)				
	RYGB	50 (66.7)	13 (72.2)	0.863 ^b^
	SG	25 (33.3)	5 (27.8)	
Postoperative time, median (Q1-Q3), days		12.0 (5.0–41.0)	2.5 (1.0–6.0)	**0.001** ^a^
EOSS group, *n* (%)				
	0–2	43 (57.3)	4 (22.2)	**0.009** ^b^
	3–4	32 (42.7)	14 (77.8)	

^a^ Mann–Whitney U test; ^b^ Pearson’s chi-square test

*BMI* Body Mass Index, *T2D *Type 2 Diabetes, *OSA *Obstructive Sleep Apnea, *MASLD *Metabolic Dysfunction-Associated Steatotic Liver Disease, *RYGB *Roux-en-Y Gastric Bypass, *SG *Sleeve Gastrectomy

The rate of overall complications increased with higher EOSS stages, from 20.1% in EOSS 2 to 38.7% in EOSS 3 and 52.6% in EOSS 4 (*p* = 0.0023). Similarly, major complication rate increased with EOSS severity, being 1.4% in EOSS 2, 8.6% in EOSS 3, and 31.6% in EOSS 4, as shown in Table 5. When grouped, patients with EOSS 3–4 had a significantly higher rate of overall (41.1% vs. 21.1%; *p* < 0.001) and major (12.5% vs. 1.8%; *p* < 0.001) complications compared to those with EOSS 0–2. Detailed description of each case with severe complication is available in Supplement 1C.

**Table 5:** Overall frequency of postoperative complications and distribution of severity according to the Clavien–Dindo classification across EOSS stages

	EOSS 0 (*n* = 3)	EOSS 1 (*n* = 13)	EOSS 2 (*n* = 207)	EOSS 3 (*n* = 93)	EOSS 4 (*n* = 19)	*p* value
Overall complications, *n* (%)	1 (33.3%)	3 (23.1%)	43 (20.1%)	36 (38.7%)	10 (52.6%)	**0.0023** ^**b**^
Minor complications, *n* (%)	1 (33.3%)	2 (15.4%)	40 (19.3%)	28 (30.1%)	4 (21%)	
Major complications, *n* (%)	-	1 (7.7%)	3 (1.4%)	8 (8.6%)	6 (31.6%)	
Clavien-Dindo classification, *n*						0.058 ^b^
I	-	1	10	7	2	
II	1	1	30	21	2	
IIIa	-	-	2	1	-	
IIIb	-	-	-	-	-	
IVa	-	1	1	3	3	
IVb	-	-	-	4	3	
V	-	-	-	-	-	

^b^Pearson’s chi-square test

### Multivariable Logistic Regression Analysis

In multivariable logistic regression analysis, EOSS was associated with higher odds of overall complications (OR = 1.90; 95% CI: 1.30–2.80; *p* = 0.001) after adjustment for age, sex, BMI, and procedure type. In contrast, age (OR = 1.01; 95% CI: 0.99–1.04; *p* = 0.236), sex (OR = 1.02; 95% CI: 0.54–1.89; *p* = 0.945), BMI (OR = 0.99; 95% CI: 0.95–1.04; *p* = 0.781), and procedure type (OR = 1.29; 95% CI: 0.75–2.27; *p* = 0.360) were not associated with overall complications (Table 6).

Given the limited number of major complications (*n* = 18), exploratory multivariable analysis for this outcome was intentionally restricted to a parsimonious model. Candidate predictors were initially selected based on clinical relevance and prior univariable analyses (Supplement 1D), followed by backward stepwise selection. The final model retained EOSS stage as the main predictor of interest and procedure type as a clinically relevant adjustment variable. In this model, higher EOSS stage remained associated with increased odds of major postoperative complications (OR 4.16; 95% CI 1.83–11.0; *p* = 0.0015). Procedure type was not significantly associated with major complications (OR 2.83; 95% CI 0.80–12.2; *p* = 0.131), although the wide confidence interval suggests limited statistical precision. Additional exploratory analyses, including alternative model specifications, multicollinearity assessment using variance inflation factors, and ROC curve analysis, are provided in the Supplementary Material (Supplement 1D).

**Table 6:** Exploratory multivariable logistic regression analyses for factors associated with overall and major 90-day postoperative complications

	OR	95% CI	*p* value	OR	95% CI	*p* value
	Overall complications			Major complications*		
Age	1.01	0.99–1.04	0.236	–		
Sex	1.02	0.54–1.89	0.945	–		
BMI	0.99	0.95–1.04	0.781	–		
Procedure type	1.29	0.75–2.27	0.360	2.83	0.80–12.2	0.131
EOSS	1.90	1.30–2.80	**0.001**	4.16	1.83–11.0	**0.0015**

*BMI* body mass index, *EOSS *Edmonton Obesity Staging System, *OR *odds ratio, *CI *confidence interval

*The model for major complications should be interpreted as exploratory due to the limited number of events

## Discussion

The present study evaluated the association between obesity severity, as assessed by the Edmonton Obesity Staging System (EOSS), and 90-day postoperative complications following metabolic and bariatric surgery (MBS). More advanced EOSS stages were associated with higher rates of both overall and major complications, demonstrating a consistent gradient between disease severity and perioperative morbidity. In multivariable analyses, more advanced EOSS stages remained associated with higher postoperative morbidity; however, these findings should be interpreted cautiously given the exploratory nature of the adjusted analyses and the limited number of major events.

The distribution of EOSS stages in our cohort revealed a high prevalence of advanced disease, with 33.4% of patients classified as EOSS 3–4. This proportion exceeds that reported in previous studies evaluating EOSS in MBS, where advanced stages ranged from 6.9% to 20.5%, and represents the highest reported prevalence of advanced EOSS in this context [19]. In the initial study by Chiappetta et al., only 12.8% of patients were classified as EOSS 3–4, with EOSS 4 being nearly absent [12]. Similarly, Skulsky et al. reported 6.9% of patients in EOSS 3 and no cases of EOSS 4 [15]. This discrepancy likely reflects temporal changes in surgical practice, with improved perioperative safety enabling the expansion of MBS indications to more complex patients [2, 3].

Notably, in our cohort, we observed a significant shift in surgical strategy across EOSS stages, with a progressive increase in the use of sleeve gastrectomy among patients with more advanced disease. This finding aligns with the growing body of evidence supporting SG in higher-risk populations, given its lower physiological impact and favorable perioperative safety profile, suggesting that surgical decision-making may be influenced by disease severity [4–6]. However, this non-random procedure allocation also introduces potential confounding by indication, as patients with greater disease severity may have preferentially undergone SG due to perceived perioperative safety advantages.

The overall postoperative complication rate in our study (27.8%) appears higher than that reported in the MBS literature. However, most postoperative events in our cohort were minor complications, with Clavien–Dindo grades I–II occurring in 22.4% of patients, whereas major complications (grades III–IV) occurred in 5.4% of patients. Notably, reoperation was required in only 1.5% of cases, and there was no 90-day mortality. These findings suggest that the elevated overall rate was largely driven by the systematic capture of lower-grade morbidity rather than by poor surgical quality or an excess of severe adverse events. Moreover, methodological differences likely further explain discrepancies with other reports, limiting formal risk-adjusted comparisons with published cohorts as most studies do not report sufficiently detailed case-mix data, including disease severity, comorbidity burden, procedural selection, and extended follow-up outcomes. Many studies focus predominantly on major complications, such as leaks, bleeding, and thromboembolic events, which may limit comparability with analyses capturing the full spectrum of postoperative morbidity [20, 21]. For instance, Chiappetta et al. reported an overall complication rate of 8.9% at 30 days; however, the higher proportion of severe complications requiring intervention compared to mild events suggests a potential bias toward capturing more clinically overt complications [14]. In contrast, studies based on detailed chart review tend to report higher complication rates. A large single-center study from the Mayo Clinic, including 2,190 patients, reported an overall complication rate of 18.9% at 30 days, with a similar distribution of minor events, particularly grade II complications (9.4%), followed by grade I (5.6%), grade III (2.1%), and grade IV (1.7%) [22]. In our cohort, although the overall complication rate was higher (27.8%), the distribution of severity was comparable, with grade II events being the most frequent (16.4%), followed by grade I (5.9%), grade III (0.9%), and grade IV (4.5%).

While the inclusion of grade I complications is sometimes debated due to their limited need for specific intervention, by definition these events represent deviations from the expected postoperative course. Even when managed conservatively, they may increase healthcare utilization, length of stay, and outpatient demand, thereby impacting both patient experience and overall cost of care. The higher proportion of grade II complications in our cohort likely reflects a more comprehensive capture of clinically relevant events requiring pharmacologic or supportive treatment. Importantly, overall complication rates should not be interpreted as equivalent to severe postoperative morbidity, as major complications requiring invasive intervention, ICU-level management, or reoperation remained relatively uncommon in this cohort. Notably, we observed a higher proportion of grade IV complications compared to the Mayo Clinic series, which is consistent with the greater disease severity of our population, as reflected by the higher prevalence of advanced EOSS stages. This finding reinforces that the observed complication profile is primarily driven by underlying disease burden rather than differences in surgical quality. Importantly, our analysis extended follow-up to 90 days, thereby further increasing sensitivity for detecting complications. In our cohort, approximately 30% of complications occurred after 30 days, highlighting the impact of extended follow-up on the observed incidence.

When stratified by EOSS, both overall and major complications increased with advancing stages. In particular, the rate of major complications reached 12.5% in patients with EOSS 3–4, compared to 1.8% in those with EOSS 0–2. These results are consistent with prior studies demonstrating higher complication rates in more advanced stages. Skulsky et al. reported major complication rates of 23.1% in EOSS 3, while Małczak et al. observed rates of 11.4% in EOSS 3 and 7.9% in EOSS 4 [15, 23].

Although absolute rates vary across studies, likely due to differences in case-mix and methodology, the consistent trend supports a strong association between EOSS severity and perioperative risk. Notably, no mortality was observed within 90 days in our cohort, despite the high prevalence of advanced EOSS stages. In contrast, previous studies evaluating EOSS and perioperative outcomes have reported low but non-zero mortality rates, ranging from 0.2% to 0.5% [14, 15, 19, 23].

Patients with major complications were more frequently classified as EOSS 3–4 and had a higher prevalence of OSA, a well-established risk factor for adverse perioperative outcomes [24]. Additionally, major complications occurred earlier in the postoperative period, suggesting a distinct clinical profile compared with minor events. In multivariable analyses, higher EOSS stages remained associated with both overall and major complications. However, these findings, particularly for major complications, should be interpreted cautiously given the exploratory nature of the adjusted analyses and the limited number of events. Nonetheless, our results are consistent with those reported by Skulsky et al., who demonstrated that higher EOSS stages were associated with increased odds of major complications after adjustment for age, sex, and BMI, while these individual variables were not independently associated with outcomes [15]. This consistency across studies reinforces the role of EOSS as an integrated measure of disease severity beyond isolated clinical factors.

Although domain-specific analyses were exploratory and are presented in the Supplementary Material, the functional domain demonstrated a stronger association with postoperative complications than the mental health domain. This finding suggests that functional impairment may represent an important component of perioperative risk in patients undergoing MBS, supporting the relevance of multidimensional obesity staging beyond isolated obesity-related medical problems. Importantly, to our knowledge, previous studies evaluating EOSS in the context of postoperative complications have not separately explored the contribution of individual EOSS domains to surgical outcomes [14, 15, 19, 23]. However, these exploratory domain-level findings should be interpreted cautiously and require validation in larger prospective cohorts. Moreover, although some exploratory analyses of individual EOSS-related conditions are presented in the Supplementary Material, these findings should be interpreted cautiously due to the limited number of events and subgroup imbalance. In particular, the inverse association observed for obesity hypoventilation syndrome likely reflects sparse-data bias and/or residual confounding rather than a true protective relationship.

These findings should also be interpreted within the broader evolution of obesity classification systems. As highlighted in recent literature, traditional BMI-based definitions fail to capture the heterogeneity and clinical impact of obesity, as they do not reflect organ dysfunction, functional limitation, or metabolic burden [9, 12]. In this context, our results support the role of EOSS as an integrated measure of obesity-related disease burden, particularly in populations with greater clinical complexity. Moreover, as newer frameworks, such as the recent Lancet Commission definition, emphasize the need for multidimensional assessment of obesity, EOSS may serve as a practical and clinically applicable tool for risk stratification and decision-making in MBS [25]. However, challenges remain regarding implementation, standardization, and integration of these staging systems into routine clinical practice.

This study has several limitations. Its retrospective design, based on EHR review, is subject to inherent limitations related to data completeness and potential information bias. In addition, because EOSS staging was assigned retrospectively from existing clinical records rather than through prospective structured assessments, some degree of misclassification may have occurred, particularly in the functional and mental health domains. Moreover, the single-center setting may limit the generalizability of the findings to other healthcare environments with different patient populations or surgical volumes. The relatively small number of major complications may have reduced the statistical power to detect independent associations, particularly for less prevalent risk factors, leading to wide confidence intervals in multivariable analyses. Likewise, the limited number of major events, particularly within procedural subgroups, precluded robust stratified, interaction, or procedure-specific sensitivity analyses according to surgical technique. Therefore, residual confounding by indication related to procedure selection cannot be fully excluded. The limited number of major events would likely have constrained the stability and interpretability of formal predictive performance analyses. Furthermore, this study was designed to assess associations rather than to develop or validate a predictive model. Finally, part of the cohort was managed during the COVID-19 pandemic, which may have influenced case selection and surgical volume, as well as the overall severity profile of patients undergoing surgery. Because these effects were dynamic and heterogeneous over time, reflecting changing lockdown policies, healthcare resource availability, and evolving disease burden, formal adjustment or period-based sensitivity analyses were unlikely to fully capture their complexity and may have introduced additional instability in this sample.

Despite these limitations, this study includes a high proportion of patients with advanced EOSS stages and is based on detailed chart review with standardized 90-day outcome assessment, allowing comprehensive capture of postoperative events beyond those typically identified in administrative datasets. The systematic application of the Clavien–Dindo classification and structured data collection using REDCap enhanced the consistency and reliability of outcome assessment. In addition, EOSS was applied using predefined criteria across clinical, functional, and mental domains, providing a more comprehensive evaluation of obesity severity than studies relying on simplified or administrative definitions, although prospective standardized domain-specific assessments would further improve classification accuracy. Future prospective multicenter studies with larger, more heterogeneous populations are needed to validate these findings and better define the independent prognostic role of EOSS. Wider and more standardized adoption of EOSS in MBS research may also improve comparability of outcomes across centers and countries, supporting more meaningful benchmarking of safety and complication rates and enhancing the real-world applicability of evidence.

## Conclusion

More advanced EOSS stages were associated with higher rates of overall and major postoperative complications within 90 days after metabolic and bariatric surgery in this cohort of patients with severe obesity and substantial disease burden. These findings suggest that EOSS may contribute to a broader assessment of perioperative risk; however, it should not be interpreted as a standalone predictor of surgical morbidity, particularly given the exploratory nature of the multivariable analyses and the relatively low number of major events in this study.

## Supplementary Information

Below is the link to the electronic supplementary material.

Supplementary Material 1 (DOCX 40.0 KB)

Supplementary Material 2 (DOCX 120 KB)

## Data Availability

All data supporting the findings of this study are available within the paper and its Supplementary Information.
