# Supplementary material for: Association Between Edmonton Obesity Staging System Severity and 90-Day Postoperative Complications After Primary Metabolic and Bariatric Surgery: A Retrospective Cohort Study
Source: Obes Surg. 2026 Jun 3;36(7):3725–35. doi: 10.1007/s11695-026-08759-2 (PMC13323407; doi:10.1007/s11695-026-08759-2)

**Supplementary Material**

**Supplement 1A. Edmonton Obesity Staging System**

| **EOSS score** | **0** | **1** | **2** | **3** | **4** |
| --- | --- | --- | --- | --- | --- |
| Definition |  |  |  |  |  |
| Clinical | NO sign of obesity-related risk  factors | Patient has obesity-related SUB-  CLINICAL risk factors (borderline hypertension, impaired  fasting glucose, elevated liver enzymes, etc.)  – OR – | Patient has ESTABLISHED  obesity-related comorbidities  requiring medical intervention (hypertension, Type 2 Diabetes (T2D),  obstructive sleep apnea,  osteoarthritis, reflux disease)  – OR – | Patient has SIGNIFICANT obesity-related end-organ damage  (myocardial infarction, heart  failure, T2D complications,  incapacitating osteoarthritis)  – OR – | Patient has SEVERE Obesity-related end-stage disease (e.g., end-stage kidney disease, cirrhosis, candidate for or previous solid organ transplantation)– OR – |
| Functional | NO psychological symptoms | MILD physical symptoms—  patient currently not requiring  medical treatment for comorbidities (dyspnea on moderate  exertion, occasional aches/  pains, fatigue, etc.)  – OR – | MODERATE functional limitations in daily activities (quality  of life beginning to be impacted)  – OR – | SIGNIFICANT functional limitations (e.g., unable to work or complete routine activities,  Reduced mobility);  SIGNIFICANT impairment  of well-being (quality of life  significantly impacted)  – OR – | SEVERE functional limitations (wheelchair dependence, limb amputation, continuous supplemental oxygen use, or bed confinement)  – OR – |
| Mental | NO functional limitations | MILD obesity –related psychological symptoms and/or mild  impairment of well-being (quality of life not impacted) | MODERATE obesity-related psychological problems (depression, eating disorders, anxiety  disorder) | SIGNIFICANT obesity-related  psychological symptoms (major  depression, suicidal ideation) | SEVERELY disabling psychological disease (suicide attempt, need for a caregiver, or current admission to a mental health institution) |

Adapted from Skulsky SL, Dang JT, Switzer NJ, Sharma AM, Karmali S, Birch DW. Higher Edmonton Obesity Staging System scores are independently associated with postoperative complications and mortality following bariatric surgery: an analysis of the MBSAQIP. Surg Endosc. 2021 Dec;35(12):7163-7173. doi: 10.1007/s00464-020-08138-7. Epub 2020 Nov 5. PMID: 33155074.

**Supplement 1B. Flowchart of patient selection and inclusion**


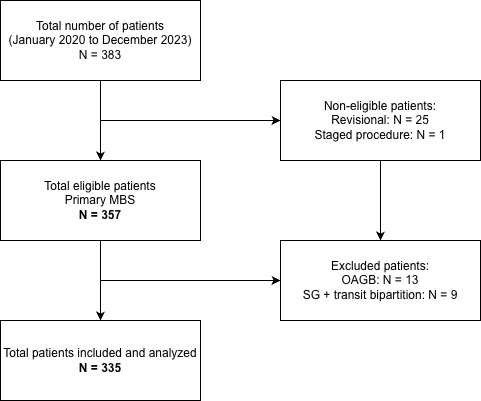


**Supplement 1C. Detailed description of each case with severe complication**

| Diagnosis | Treatment | Day | Clavien-Dindo |
| --- | --- | --- | --- |
| EOSS 0 – 2 (n = 4) |  |  | |
| Bleeding from gastrojejunal anastomosis | Endoscopic treatment, without hemodynamic instability and without need for transfusion. | 6 | IIIa |
| Gastrojejunal stenosis | Endoscopic dilation | 42 | IIIa |
| Hemorrhagic shock from melena | Volume resuscitation, blood transfusion, and ICU monitoring, without need for procedural intervention. | 3 | IVa |
| Bleeding from gastrojejunal anastomosis | Endoscopic treatment, with endotracheal intubation for airway protection and ICU monitoring, without hemorrhagic shock. | 1 | IVa |
| EOSS 3 – 4 (n = 14) |  |  | |
| Gastrojejunal stenosis | Endoscopic dilation | 29 | IIIa |
| Pulmonary embolism | Anticoagulation and supplemental oxygen therapy in the ICU. | 2 | IVa |
| Acute kidney injury on chronic kidney disease (AKI on CKD) | Hyperkalemia requiring ICU monitoring, without the need for urgent dialysis. | 2 | IVa |
| Intrabdominal bleeding complicated with acute kidney injury | Conservative management with ICU monitoring, without the need for transfusion. | 6 | IVa |
| Tension pneumothorax | Intraoperative hemodynamic instability, with ICU monitoring following chest tube drainage. | 0 | IVa |
| Pneumonia with dyspnea | Supplemental oxygen therapy and antibiotic treatment in the ICU. | 3 | IVa |
| Hypertensive crisis | Requirement for vasoactive therapy in the ICU (nitroprusside). | 1 | IVa |
| Acute pulmonary edema due to COVID-19–related complications | Medical management in the ICU | 23 | IVb |
| Septic shock due to inadvertent small bowel injury | Reoperation with laparoscopy and ICU monitoring | 1 | IVb |
| Acute renal graft dysfunction due to dehydration following recurrent vomiting | Medical management in the ICU without the need for hemodialysis, complicated by bloodstream infection | 58 | IVb |
| Hemorrhagic shock due to intrabdominal bleeding | Laparoscopic reoperation with clipping of the vascular stump at the staple line, followed by ICU monitoring with vasoactive support, complicated by acute kidney injury. | 0 | IVb |
| Decompensated heart failure | ICU admission for vasoactive support with norepinephrine and dobutamine, complicated by (AKI on CKD) without the need for hemodialysis. | 1 | IVb |
| Hemorrhagic shock due to intrabdominal bleeding | Laparoscopic reoperation with clipping of the vascular stump at the staple line, followed by ICU monitoring with vasoactive support, complicated by acute kidney injury. | 0 | IVb |
| Septic shock due to superficial infection of a pressure ulcer | Surgical debridement and medical management in the ICU | 4 | IVb |

ICU – Intensive Care Unit

**Supplement 1D.**

After univariate analyses, two separate multivariable modeling strategies were applied to address potential collinearity between EOSS and its individual components for overall and major complications. These analyses along with variance inflation factors (VIFs) are presented as follows:

**Univariate logistic regression analyses for factors associated with overall 90-day postoperative complications**

|  | **OR** | **CI 95%** | **p value** |
| --- | --- | --- | --- |
| Age | 1.02 | 1.00 – 1.04 | 0.088 |
| Sex | 1.34 | 0.73 – 2.42 | 0.334 |
| BMI | 0.98 | 0.95 – 1.02 | 0.347 |
| T2D | 1.43 | 0.88 – 2.31 | 0.147 |
| Insulin use | 2.33 | 1.12 – 4.85 | **0.023** |
| Hypertension | 1.58 | 0.94 – 2.72 | 0.088 |
| Dyslipidemia | 1.36 | 0.83 – 2.21 | 0.220 |
| MASLD | 0.59 | 0.34 – 1.04 | 0.064 |
| OSA | 1.24 | 0.74 – 2.05 | 0.410 |
| Hypoventilation | 0.37 | 0.12 – 0.91 | **0.047** |
| Incisional hernia | 0.50 | 0.11 – 1.57 | 0.288 |
| Previous VTE | 1.66 | 0.64 – 4.08 | 0.279 |
| Pulmonary hypertension | 1.30 | 0.06 – 13.77 | 0.829 |
| Previous MI | 3.38 | 0.88 – 13.93 | 0.074 |
| Heart failure | 3.60 | 1.30 – 10.35 | **0.014** |
| Use of anticoagulation | 2.02 | 0.58 – 6.52 | 0.241 |
| Use of corticosteroids | 8.47 | 1.07 – 172.56 | 0.066 |
| Bariatric procedure | 0.98 | 0.59 – 1.65 | 0.938 |
| EOSS staging | 1.89 | 1.33 – 2.73 | **< 0.001** |
| EOSS – medical domain | 1.75 | 1.22 – 2.53 | **0.003** |
| EOSS – functional domain | 1.50 | 1.18 – 1.92 | **0.001** |
| EOSS – mental domain | 1.13 | 0.89 – 1.42 | 0.312 |
| EOSS 3 – 4 | 2.61 | 1.59 – 4.29 | **< 0.001** |

^BMI: body mass index; T2D: type 2 diabetes; OSA: obstructive sleep apnea; MASLD: metabolic dysfunction-associated steatotic liver disease; VTE: venous thromboembolism; MI: myocardial infarction; EOSS: edmonton obesity staging system^

Note: The inverse association observed for obesity hypoventilation syndrome should be interpreted with considerable caution. Given the small number of events and subgroup imbalance, this finding likely reflects sparse-data bias, residual confounding, and/or procedure-selection effects rather than a true protective association

**Univariate logistic regression analyses for factors associated with major 90-day postoperative complications**

|  | **OR** | **CI 95%** | **p value** |
| --- | --- | --- | --- |
| Age | 1.03 | 0.99 – 1.08 | 0.163 |
| Gender | 3.03 | 0.96 – 9.30 | 0.052 |
| BMI | 0.97 | 0.89 – 1.05 | 0.466 |
| T2D | 2.29 | 0.80 – 7.16 | 0.134 |
| Insulin use | 3.38 | 0.89 – 14.83 | 0.084 |
| Hypertension | 3.54 | 0.91 – 23.54 | 0.110 |
| Dyslipidemia | 1.98 | 0.70 – 5.76 | 0.197 |
| MASLD | 0.51 | 0.17 – 1.58 | 0.230 |
| OSA | 5.50 | 1.88 – 17.69 | **0.003** |
| Hypoventilation | 1.04 | 0.05 – 7.64 | 0.970 |
| Incisional hernia | 2.15 | 0.10 – 23.70 | 0.542 |
| Previous VTE | 1.44 | 0.20 – 6.94 | 0.674 |
| Previous MI | 7.30 | 1.12 – 59.18 | **0.038** |
| Heart failure | 6.83 | 1.61 – 31.01 | **0.009** |
| Use of anticoagulation | 6.20 | 0.95 – 50.36 | 0.057 |
| Use of corticosteroids | 7.88 | 0.71 – 175.86 | 0.100 |
| Bariatric procedure | 1.30 | 0.44 – 4.42 | 0.651 |
| EOSS staging | 3.24 | 1.55 – 7.46 | **0.003** |
| EOSS – medical domain | 2.73 | 1.33 – 6.01 | **0.008** |
| EOSS – functional domain | 1.97 | 1.22 – 3.32 | **0.007** |
| EOSS – mental domain | 1.14 | 0.67 – 1.89 | 0.621 |
| EOSS 3 – 4 | 4.70 | 1.52 – 17.83 | **0.012** |

^BMI: body mass index; T2D: type 2 diabetes; OSA: obstructive sleep apnea; MASLD: metabolic dysfunction-associated steatotic liver disease; VTE: venous thromboembolism; MI: myocardial infarction; EOSS: edmonton obesity staging system^

**Variance Inflation Factors**

| **Variable** | **VIF** |
| --- | --- |
| EOSS | 1.27 |
| Insulin use | 1.11 |
| Heart failure | 1.19 |
| Hypoventilation | 1.09 |
| Previous MI | 1.16 |
| Age | 1.21 |
| BMI | 1.18 |
| Sex | 1.07 |
| Procedure type | 1.09 |

^BMI: body mass index; MI: myocardial infarction; EOSS: edmonton obesity staging system^

**Multivariable Models for Overall Complications**

|  | **Model A (EOSS)** | |  | **Model B (obesity-related medical problems)** | |
| --- | --- | --- | --- | --- | --- |
| **Variable** | **OR (95% CI)** | **p-value** |  | **OR (95% CI)** | **p-value** |
| Insulin use | — | — |  | 2.38 (1.09–5.24) | 0.029 |
| Heart failure | — | — |  | 3.64 (0.85–17.5) | 0.086 |
| Hypoventilation | — | — |  | 0.19 (0.03–0.78) | 0.044 |
| Previous MI | — | — |  | 1.00 (0.14–6.27) | 0.999 |
| Age | 1.01 (0.99–1.04) | 0.236 |  | 1.01 (0.97–1.05) | 0.726 |
| BMI | 0.99 (0.95–1.04) | 0.781 |  | 1.04 (0.97–1.11) | 0.256 |
| Sex | 1.02 (0.54–1.89) | 0.945 |  | 1.21 (0.50–2.82) | 0.670 |
| Procedure | 1.29 (0.75–2.27) | 0.360 |  | 1.24 (0.55–2.88) | 0.614 |
| EOSS | 1.90 (1.30–2.80) | 0.001 |  | — | — |

^BMI: body mass index; MI: myocardial infarction; EOSS: edmonton obesity staging system^

**Multivariable Models for Major Complications**

|  | **Model A (EOSS)** | |  | **Model B (obesity-related medical problems)** | |
| --- | --- | --- | --- | --- | --- |
| **Variable** | **OR (95% CI)** | **p-value** |  | **OR (95% CI)** | **p-value** |
| Insulin use | — | — |  | 2.43 (0.48–12.1) | 0.298 |
| Heart failure | — | — |  | 3.39 (0.36–32.6) | 0.285 |
| Hypoventilation | — | — |  | — (unstable) | 0.995 |
| Previous MI | — | — |  | 2.64 (0.14–50.7) | 0.537 |
| Age | 1.03 (0.97–1.09) | 0.282 |  | 1.07 (0.96–1.18) | 0.222 |
| BMI | 0.99 (0.90–1.10) | 0.821 |  | 0.99 (0.85–1.16) | 0.868 |
| Sex | 1.87 (0.52–6.74) | 0.328 |  | 1.46 (0.22–9.70) | 0.696 |
| Procedure | 3.61 (0.85–15.3) | 0.082 |  | 1.59 (0.23–10.9) | 0.639 |
| EOSS | 3.71 (1.45–9.54) | 0.006 |  | — | — |

^BMI: body mass index; MI: myocardial infarction; EOSS: edmonton obesity staging system^

Because only 18 major complications occurred, the exploratory multivariable analysis was intentionally restricted to a parsimonious model to reduce overfitting risk. Clinically relevant candidate predictors were initially considered, followed by backward selection. The final model retained EOSS stage and procedure type. Model discrimination was evaluated by the area under the ROC curve, showing moderate discrimination, with an AUC of 0.74. Given the limited number of major complications (n = 18), these findings should be interpreted cautiously, particularly for procedure type, where the wide confidence interval suggests limited statistical precision.

**Exploratory multivariable logistic regression analyses for factors associated with major 90-day postoperative complications**

|  |  | **OR** | **95% CI** | **p value** |
| --- | --- | --- | --- | --- |
|  |  | Major complications* | | |
| Procedure type |  | 2.83 | 0.80 – 12.2 | 0.131 |
| EOSS |  | 4.16 | 1.83 – 11.0 | **0.0015** |

*The model for major complications should be interpreted as exploratory due to the limited number of events.


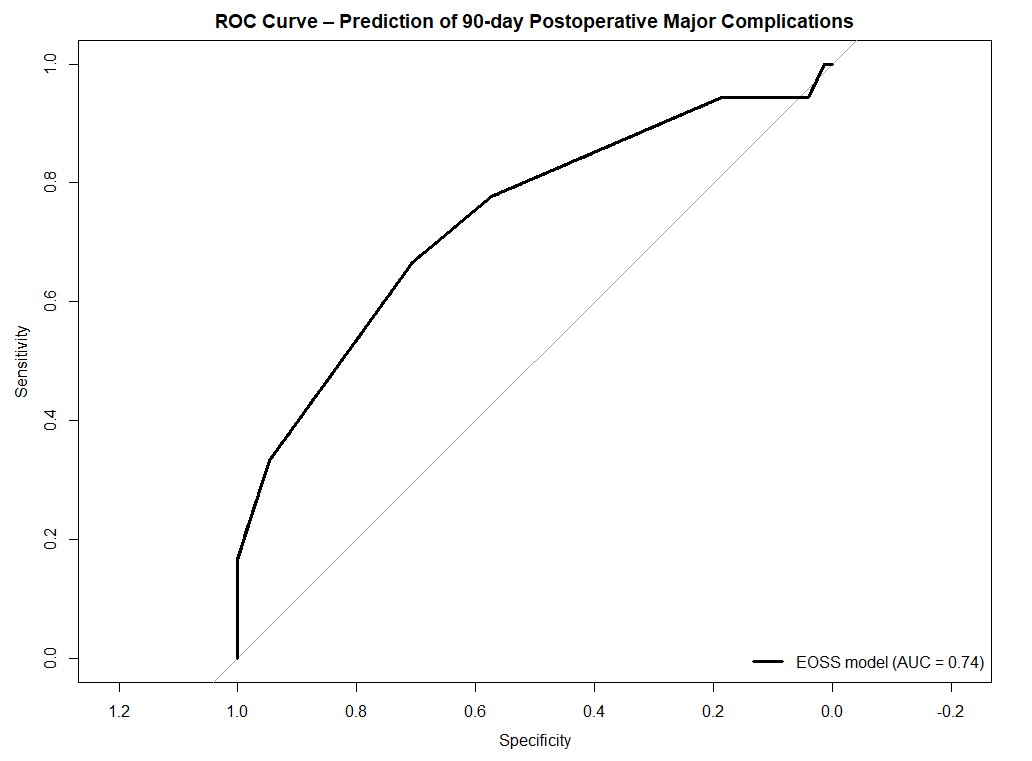

Supplement: Supplementary file 2 — Supplementary Material 2 (DOCX 120 KB) [file 11695_2026_8759_MOESM2_ESM.docx]
